# Oxidized LDL Induces Alternative Macrophage Phenotype through Activation of CD36 and PAFR

**DOI:** 10.1155/2013/198193

**Published:** 2013-08-25

**Authors:** Francisco J. Rios, Marianna M. Koga, Mateus Pecenin, Matheus Ferracini, Magnus Gidlund, S. Jancar

**Affiliations:** ^1^Department of Immunology, Institute of Biomedical Sciences, University of Sao Paulo, Avenida Professor Lineu Prestes 1730, ICB IV—Sala 140/146, 05508-900 Sao Paulo, SP, Brazil; ^2^BHF-Glasgow Cardiovascular Research Centre, Institute of Cardiovascular and Medical Sciences, University of Glasgow, Glasgow G12 8TA, UK

## Abstract

OxLDL is recognized by macrophage scavenger receptors, including CD36; we have recently found that Platelet-Activating Factor Receptor (PAFR) is also involved. Since PAFR in macrophages is associated with suppressor function, we examined the effect of oxLDL on macrophage phenotype. It was found that the presence of oxLDL during macrophage differentiation induced high mRNA levels to IL-10, mannose receptor, PPAR**γ** and arginase-1 and low levels of IL-12 and iNOS. When human THP-1 macrophages were pre-treated with oxLDL then stimulated with LPS, the production of IL-10 and TGF-**β** significantly increased, whereas that of IL-6 and IL-8 decreased. In murine TG-elicited macrophages, this protocol significantly reduced NO, iNOS and COX2 expression. Thus, oxLDL induced macrophage differentiation and activation towards the alternatively activated M2-phenotype. In murine macrophages, oxLDL induced TGF-**β**, arginase-1 and IL-10 mRNA expression, which were significantly reduced by pre-treatment with PAFR antagonists (WEB and CV) or with antibodies to CD36. The mRNA expression of IL-12, RANTES and CXCL2 were not affected. We showed that this profile of macrophage activation is dependent on the engagement of both CD36 and PAFR. We conclude that oxLDL induces alternative macrophage activation by mechanisms involving CD36 and PAFR.

## 1. Introduction

 The stimulation of monocytes/macrophages by modified low density lipoprotein (LDL), such as oxidized LDL (oxLDL), is an early event in atherosclerosis development. These macrophages accumulate in the subendothelial space and differentiate into foam cells [1], which contribute to a chronic inflammatory response in the arterial wall and atherosclerotic plaque progression [2]. Several pattern recognition receptors are involved in oxLDL recognition by macrophages; CD36 is one of the most studied.

Macrophages can acquire distinct phenotypes according to stimuli from the microenvironment. M1 or classically activated macrophages are induced by Th1 cytokines and exhibit high microbicidal activity and induce inflammation. In contrast, M2 or alternatively activated macrophages are induced by Th2 cytokines and contribute to the resolution of inflammation and tissue remodeling [3]. The atherosclerotic plaque provides a complex microenvironment for macrophages, and both populations, M1 and M2, have been found in human lesions [4]. It has been demonstrated that M1 macrophages are predominant in regions prone to rupture, while M2 macrophages are mostly detected in the adventitia and in stable plaque areas [5]. However, it is not known which role macrophages with such opposing functions play in the progression of atherosclerosis. M2 macrophages express high levels of CD36 and SR-A1 and are thus able to efficiently take up oxLDL and are more prone to differentiating into foam cells [6]. Macrophages also express receptors for platelet-activating factor (PAFR), and our previous work suggested that PAFR works in conjunction with CD36 for optimal oxLDL uptake and cytokine gene expression [7]. Moreover, preferential production of IL-10 over IL-12 was observed (submitted article). 

In the present study, we investigated the effect of oxLDL on macrophage differentiation, activation, and phenotype and the role played by CD36 and PAFR. We found that oxLDL increases the expression of alternative activation markers and that both CD36 and PAFR are involved.

## 2. Methods

### 2.1. Purification and Oxidation of LDL

The study was approved by the ethics committee of the Institute of Biomedical Sciences, University of São Paulo. Plasma was obtained from normolipidemic volunteers and treated with benzamidine (2 mM), gentamicin (0.5%), chloramphenicol (0.25%), phenyl-methyl-sulfonyl-fluoride (PMSF) (0.5 mM), and aprotinin (0.1 units/mL). LDL (1.019–1.063 g/mL) was isolated by sequential ultracentrifugation at 100,000 g at 4°C, using a P90AT-0132 rotor (CP70MX ultracentrifuge; Hitachi Koki Co., Ltd., Tokyo, Japan). LDL was dialyzed at 4°C against PBS (pH 7.4) with 1 mM EDTA, filtered (0.22 *μ*m), and stored at 4°C. The protein concentration was determined by the BCA kit (Pierce, USA). Oxidized LDL (oxLDL) was obtained by incubation of LDL (2 mg/mL) with 20 *μ*M CuSO_4_ for 18 h at 37°C. The oxidation of both protocols was terminated by the addition of 0.5 mM EDTA.

### 2.2. Human THP-1 Monocytic Cells

The monocytic line THP-1 was cultured in RPMI-1640 medium supplemented with 5% (v/v) fetal bovine serum (FBS), 100 U/mL penicillin, 100 *μ*g/mL streptomycin, 2 mM L-glutamine, 15 mM HEPES, and 11 mM sodium bicarbonate. Cell cultures were maintained in a humidified atmosphere containing 5% CO_2_ at 37°C. The differentiation of THP-1 monocytes into macrophages was induced by 150 nM phorbol 12-myristate-13-acetate (PMA) for 24 h. Nonadherent cells were removed by aspiration of the supernatant followed by replacement with fresh medium. The experiments proceeded for 24 h according to the protocol.

### 2.3. Culture of Murine Macrophages

Male C57Bl/6 7–10 weeks old mice were acquired from the Department of Immunology Animal Facility at the University of São Paulo and kept in microisolator cages under specific pathogen-free conditions. Animal care and research protocols were in accordance with the principles and guidelines adopted by the Brazilian College of Animal Experimentation (COBEA) and approved by the Biomedical Sciences Institute/USP-Ethical Committee for Animal Research (CEEA). Peritoneal exudate cells were obtained by lavage of the peritoneal cavity 4 days after injection of 1 mL of 3% thioglycolate medium. The fluid lavage was centrifuged (100 ×g, 10 min, 4°C). The cellular concentration was adjusted to 2 × 10^6^ cells/mL with RPMI 1640 medium, supplemented with 5% FCS, streptomycin (100 *μ*g/mL), penicillin (60 U/mL), sodium bicarbonate (11 mM), l-glutamine (2 mM), and HEPES (20 mM), referred to hereafter as RPMI/FBS. Cells were left to adhere on microplates for 2 h at 37°C in a 5% CO_2_. Nonadherent cells were removed by aspiration of the supernatant and replacement with fresh medium. The experiments proceeded according to the protocol.

Bone marrow-derived macrophages (BMDM) were obtained as previously described by Davies and Gordon [8], with minor modifications. In brief, femurs were flushed with DMEM medium containing 2 mM L-glutamine, 100 U/mL penicillin G, and 100 mg/mL streptomycin (all from Gibco, Long Island, NY, USA), using a 26G × 1/2′′ needle. Cells were grown in DMEM containing 20% L-929 cell-conditioned medium (LCM) and 15% heat-inactivated fetal calf serum (FCS), incubated at 37°C in 5% CO_2_. On day 3, fresh DMEM with LCM was added. A monolayer of macrophages was scrapped on day 6 (96% of the cells were positive for CD11b and F4/80). Macrophages were cultured in DMEM with 5% FCS for one day before further experiments.

### 2.4. Quantification of Nitric Oxide

NO production was assessed by nitrite production in culture supernatants using the Griess reaction. Briefly, culture supernatants were incubated with the Griess reagent (0.1% naphthylethylenediamine dihydrochloride and 1% sulfanilamide in 2.5% phosphoric acid, v/v) at room temperature for 10 min. Absorbance was determined using a Dinatech microplate reader at 540 nm. A standard curve of sodium nitrite was used to determine the concentration.

### 2.5. Western Blot for iNOS and COX-2 Expression

Thioglycolate-elicited macrophages were treated with different concentrations of LDL or oxLDL for 24 h and then stimulated with LPS (1 *μ*g/mL). Cells were washed with cold PBS, and lysates were obtained in lysis buffer (10% Nonidet P-40, 150 mM NaCl, 10 mM Tris-HCl, pH 7.6, and 2 mM 0.1% SDS) supplemented with a protease inhibitor cocktail (Sigma-Aldrich, Saint Louis, MO, USA) and phosphatase inhibitors (10 mM sodium fluoride and 1 mM sodium orthovanadate). Protein concentrations were determined using the Pierce BCA Protein Assay Kit (Thermo Scientific, Rockford, IL, USA). Equal amounts of proteins were separated by 10% SDS-PAGE, transferred to a Hybond nitrocellulose membrane (GE Healthcare, NJ, USA), and incubated with rabbit-anti-COX-2 or rabbit-anti-iNOS (both from Cayman Chemical, Ann Arbor, MI, USA), and with mouse anti-*β*-actin (Sigma-Aldrich, Saint Louis, MO, USA). As secondary antibodies, we used antirabbit IgG-HPR (1 : 2000) and antimouse-HRP (1 : 2000) (Cell Signaling Technology, Beverly, MA, USA). Expression was visualized using SuperSignal West Pico Chemiluminescent Substrate (Thermo Scientific, Rockford, IL, USA). The resulting autoradiograms were analyzed with the AlphaEaseFC software V3.2 beta (Alpha Innotech, San Leandro, CA, USA).

### 2.6. MTT Assay

The mitochondrial-dependent reduction of methylthiazolyldiphenyl-tetrazolium bromide (MTT) into insoluble formazan crystals was used to evaluate cell viability. Briefly, 500 mg/mL of MTT in RPMI was added to the cells after treatment. Cells were incubated for 3 h at 37°C in a 5% CO_2_ atmosphere. Next, a solution of 10% SDS in 0.01 M HCl was added to the cells to dissolve the crystals, and the absorbance was measured after 14 h using a Dynatech microplate reader at 570 nm.

### 2.7. Real-Time RT-PCR

Cells were harvested at various time points, and total RNA was isolated using the Trizol reagent (Invitrogen, Carlsbad, CA, USA) according to the manufacturer's instructions. cDNA was generated from total RNA using the RevertAid First Strand cDNA Synthesis Kit (Thermo Scientific Fermentas, Vilnius, Lithuania). Real-time PCR was performed with the Stratagene MxPro3005PTM QPCR Systems (Santa Clara, CA, USA), using SYBR Green (SYBR Green Master Mix, Applied Biosystems, Warrington, UK) and specific primers for *IL-10, MR, Arg-1, PPAR-*γ*, iNOS, IL-12p40, TGF-*β*1, RANTES, *and *GAPDH *(Table 1). Relative gene expression was calculated by the 2^−ΔΔCT^ method as previously described [9]. Data are shown as the fold change in expression of the target gene relative to the internal control gene (*GAPDH*).

### 2.8. Detection of Cytokines

Human IL-6, IL-8, IL-10, TGF-*β*, IL-1*β*, and TNF-*α* and murine IL-10 and IL-12p40 production were determined by ELISA (ELISA Kits, BD Biosciences, San Diego, CA, USA) according to the manufacturer's specifications.

## 3. Results and Discussion

### 3.1. oxLDL Preferentially Increases LPS-Induced Anti-Inflammatory Cytokines

**Figure 1:**
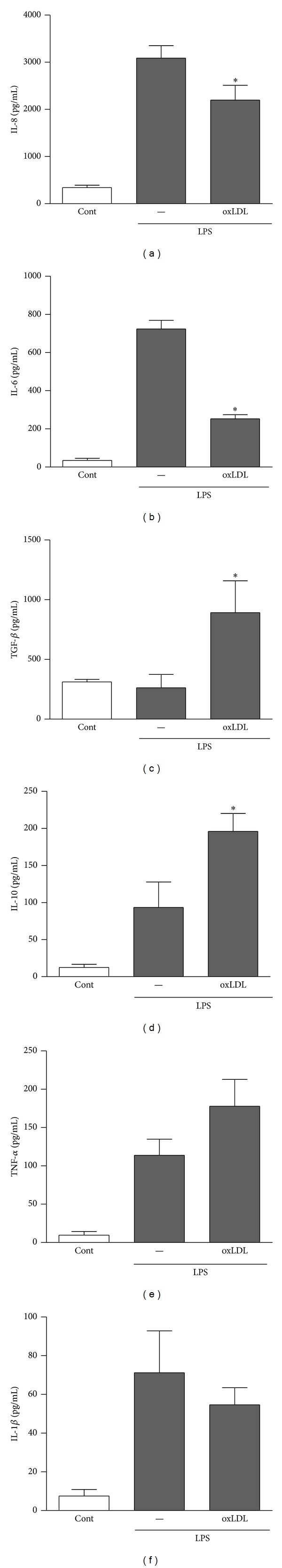
oxLDL preferentially increases LPS-induced anti-inflammatory cytokines. THP-1 monocytes were differentiated into macrophages with PMA, followed by treatment with oxLDL (20 *μ*g/mL) for 24 h, and then stimulated with LPS (100 ng/mL) for an additional 24 h. Cytokines concentration in the supernatant was measured by ELISA. Data are presented as mean ± SEM of four independent experiments. *P < 0.05  versus LPS-stimulated cells.

Oxidized LDL was obtained according to a previous publication and presented high values for both negative charge and TBARS compared to non-modified LDL [10]. Human monocytic THP-1 cells were differentiated into macrophages using PMA, followed by treatment with oxLDL (20 *μ*g/mL); 24 h later, they were stimulated with LPS (100 ng/mL) for an additional 24 h. We found that pretreatment with oxLDL decreased the LPS-induced production of IL-8 and IL-6 (29% and 34% inhibition, resp.), potentiated the production of IL-10 (2-fold increase) and TGF-*β* (3-fold increase), and did not significantly affect TNF-*α* and IL-1*β* production (Figure 1). Although pretreatment with oxLDL did not significantly affect the LPS-induced production of TNF*α* and IL-1*β*, the fact that it decreased IL-6 and IL-8 and increased IL-10 and TGF-*β* production suggests that oxLDL stimulates macrophages towards an alternative activation phenotype.

It is known that depending on the degree of LDL oxidation, different products are formed, and distinct biological effects have been attributed to the LDL preparations subjected to high and low oxidation [11–13]. Here, we used oxLDL with high oxidative degree. In this situation, phospholipids, triacylglycerol, and cholesterol esters are transformed into hydroperoxides which react with ApoB-100, resulting in modification and fragmentation of amino acid side chains [14, 15]. 

### 3.2. oxLDL Inhibits LPS-Induced NO, iNOS, and COX-2

**Figure 2:**
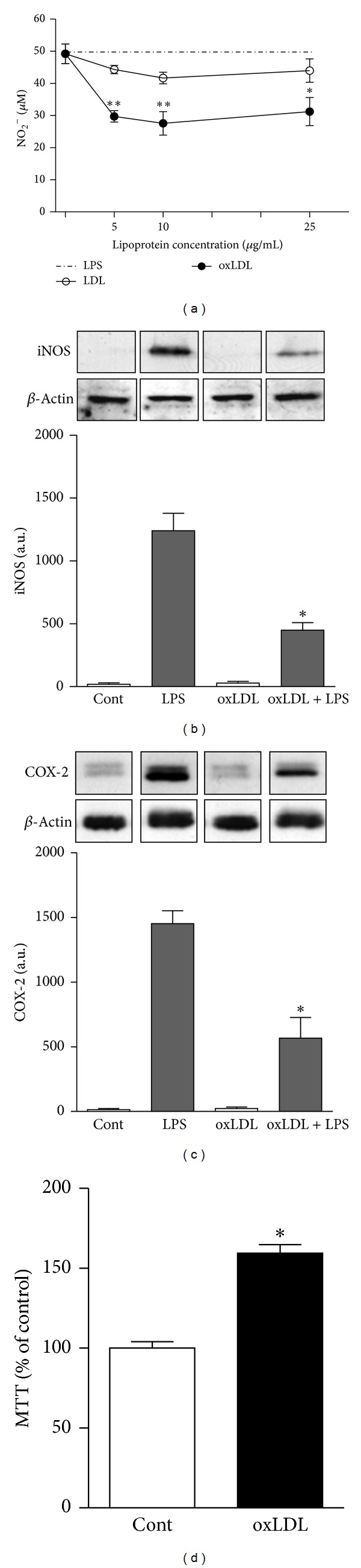
oxLDL treatment inhibits LPS-induced NO, iNOS, and COX-2. Thioglycolate-elicited murine macrophages were treated with different concentrations of LDL or oxLDL for 24 h and then stimulated with LPS (1 *μ*g/mL) for an additional 24 h. (a) Nitric oxide production was analyzed by the Griess assay. (b) iNOS and COX-2 expressions were analyzed by western blot, and the protein expression was quantified by AlphaEase FC software, V3.2 beta (Alpha Innotech). The autoradiographs show one representative experiment, *P < 0.05  versus LPS stimulated cells. (c) Cell viability was measured by MTT assay, *P < 0.05 comparing oxLDL-treated with the nontreated cells. Data are presented as mean ± SEM of six independent experiments.

We next examined the effect of oxLDL on thioglycolate-elicited murine macrophages that already express a pro-inflammatory phenotype. Macrophages were treated with different concentrations of oxLDL for 24 h and then stimulated with LPS (1 *μ*g/mL) for an additional 24 h.  Figure 2(a) shows that oxLDL inhibits nitric oxide (NO) production induced by LPS in a dose-dependent manner. No effect was observed when nonoxidized LDL was used. Treatment with oxLDL also inhibited the expression of iNOS and COX-2 induced by LPS stimulation (52% and 55% for iNOS and COX-2, resp.) (Figures 2(b) and 2(c)). The inhibitory effects of oxLDL were not related to a decrease in cell viability, evaluated by measuring mitochondrial activity by the MTT assay, which was actually increased in oxLDL-treated macrophages (Figure 2(d)).

Although oxLDL particles have been associated with proinflammatory mechanisms related to the development of atherosclerosis [2], our data indicate that oxLDL increases anti-inflammatory and reduces pro-inflammatory markers induced by LPS, favoring macrophage differentiation toward the M2 phenotype. Compounds present in the oxLDL particle, such as sphingosine 1-phosphate (S1P) and oxidized 1-palmitoyl-2-arachidonoyl-sn-glycero-3-phosphocholine (oxPAPC) have been shown to inhibit TLR2 and TLR4 activation, respectively [16, 17]. The complexity involved in this mechanism could be explained by interactions between different compounds formed after the oxidative process with different receptors present on macrophages.

### 3.3. The Presence of oxLDL during Macrophage Differentiation Induces the M2 Phenotype

**Figure 3:**
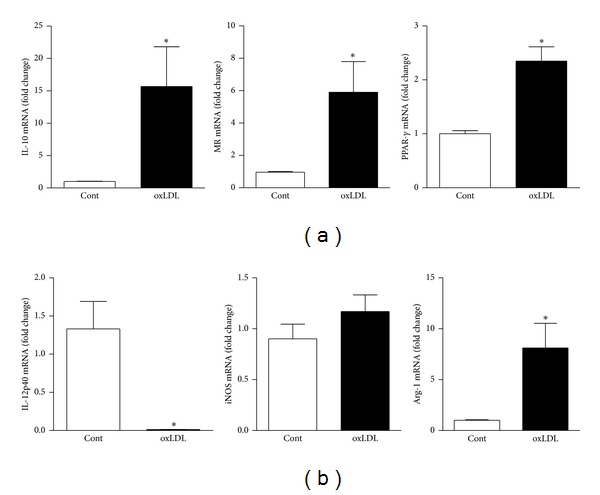
oxLDL induces macrophages differentiation towards M2 phenotype. Murine bone marrow-derived cells were differentiated to macrophages (BMDM) with L929 supernatant for six days in the presence of oxLDL. Cells were treated with oxLDL (20 *μ*g/mL) on the first day of culture and supplemented every two days. The mRNA expression of IL-10, arginase-1 (Arg-1), mannose receptor (MR), PPAR*γ*, IL-12p40, and iNOS was assessed by real-time PCR. Data are presented as mean ± SEM of independent experiments and expressed in fold change. *P < 0.05 comparing oxLDL-treated with nontreated cells.

Murine bone marrow-derived cells were differentiated to macrophages (BMDM) with L929 supernatant for six days in the presence of oxLDL. Cells were treated with oxLDL (20 *μ*g/mL) on the first day of culture and supplemented every two days. We found that macrophages treated with oxLDL expressed high levels of mRNA for the M2 macrophage markers IL-10, arginase-1 (Arg-1), mannose receptor (MR), and PPAR*γ* and decreased expression of IL-12 mRNA with no effect on iNOS mRNA (Figure 3). This indicates that the presence of oxLDL during macrophage differentiation shifts the phenotype toward the M2 profile.

### 3.4. Engagement of PAFR and CD36 is Required for the oxLDL-Induced M2 Phenotype

**Figure 4:**
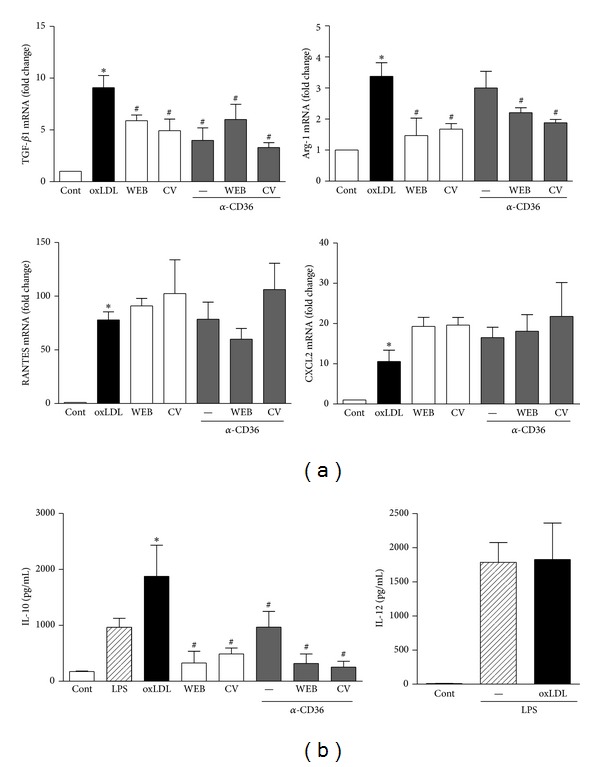
Engagement of PAFR and CD36 is required for the oxLDL-induced M2 phenotype. Murine BMDM were pretreated with PAFR antagonists WEB2086 (50 *μ*M) or CV3988 (10 *μ*M) alone or in combination with blocker antibody to CD36 (1 : 500) for 30 min and then treated with oxLDL (20 *μ*g/mL). (a) The mRNA expression of TGF-*β*, Arg-1, RANTES and CXCL2 was analyzed by real-time PCR after 5 h. (b) BMDM were treated overnight with oxLDL and then stimulated with LPS (10 ng/mL). IL-10 and IL-12 production was assessed in the supernatants by ELISA assays. *P < 0.05 comparing oxLDL-treated with nontreated cells and ^#^ *P* < 0.05  versus oxLDL-treated cells.

In a previous study, we found that costimulation of PAFR and CD36 is needed for optimal macrophage activation induced by oxLDL [7]. Here, we investigated if both receptors are involved in the induction of the M2 phenotype. Murine BMDM were treated with PAFR antagonists (WEB2086 or CV3988) alone or in combination with blocking antibody to CD36 for 30 min and then treated with oxLDL (20 *μ*g/mL) for 5 h.  Figure 4(a) shows that oxLDL induced the expression of TGF-*β* and Arg-1 mRNA and that this was reversed by treatment with PAFR antagonists (TGF-*β*: 36% and 45% inhibition; Arg1: 57% and 50% inhibition for WEB and CV, resp.). Blockage of CD36 reduced only the mRNA expression of TGF-*β* (56%). Simultaneous blockage of CD36 and PAFR did not further reduce Arg-1 and TGF-*β* mRNA expression. The oxLDL also induced the expression of RANTES and CXCL2, but this was not affected by treatment with the PAFR antagonists or CD36 blocking antibody. Next, we examined the requirement of PAFR and CD36 for IL-10 and IL-12 production. To ensure the production of detectable levels of these cytokines, the cells were activated with LPS. BMDM were pretreated with the CD36 blocking antibody alone, or in combination with the PAFR antagonists 30 min before overnight stimulation with oxLDL (20 *μ*g/mL) followed by activation with LPS (10 ng/mL).  Figure 4(b) shows that oxLDL increased the LPS-induced production of IL-10 but did not affect IL-12. Furthermore, the IL-10 concentration was strongly reduced by the PAFR antagonists WEB and CV and by the CD36 blocking antibody.

Our results show that both CD36 and PAFR are involved in IL-10 production. Although several receptors may be involved in oxLDL recognition [18], the upregulation of IL-10 induced by oxLDL depends mainly on CD36 and PAFR since, in the present study, the production of this cytokine was almost completely blocked by treatment with PAFR antagonists and antibodies to CD36. It has been described that oxLDL induces Arginase expression and activity in macrophages and in endothelial cells [19, 20]. We showed here that PAFR antagonists decreased Arg-1 and TGF-*β*1 mRNA expression induced by oxLDL, indicating that PAFR activation is required for the induction of alternative activation markers in macrophages. Atherosclerosis is characterized by a chronic inflammatory reaction in the arterial wall, and both forms of activated macrophages, classical and alternative, have been found in atherosclerotic lesions [4]. M1 macrophages are mostly present in areas prone to rupture, while M2 macrophages are found in the adventitia, and both M1 and M2 are present in the fibrous cap [5]. However, the real function of each macrophage population still needs to be elucidated. In the atherosclerotic plaque, there are different forms of modified LDL. Here, we found that LDL with a high degree of oxidation increased anti-inflammatory markers in a PAFR- and CD36-dependent manner. We do not exclude the idea that additional mechanisms may contribute to oxLDL-induced macrophage differentiation into the M1 or M2 phenotype. It can be speculated that the presence of M2 macrophages in the plaque is an attempt of the organism to control the inflammatory response; in this case, the PAFR would be involved in atheroprotection. However, M2 macrophages express more CD36 [4, 6] and are more likely to become foam cells, which contribute to the pathophysiology of atherosclerosis [21]. Moreover, these cells are more sensitive to death in a high lipid environment, such as the lipid core of the atherosclerotic plaque [6].

## 4. Conclusions

In the present study, we demonstrated that oxLDL induces macrophage differentiation towards an alternative phenotype and that this requires the engagement of PAFR and CD36. As the real function of M2 macrophages in the development of atherosclerosis remains unclear, *in vivo* studies using PAFR antagonists are needed before suggesting their use as a therapeutic approach for the treatment of atherosclerosis.

## Figures and Tables

**Table 1:** List of primer sequences used for real-time RT-PCR analysis in this study.

Name	Forward	Reverse
IL-10	5′-CAGAGCCACATGCTCCTAGA-3′	5′-TGTCCAGCTGGTCCTTTGTT-3′
MR	5′-GATATGAAGCCATGTACTCCTTACTGG-3′	5′-GGCAGAGGTGCAGTCTGCAT-3′
Arg-1	5′-TTCTCAAAAGGACAGCCTCG-3′	5′-AGCTCTTCATTGGCTTTCCC-3′
PPAR-*γ*	5′-TCCTGTAAAAGCCCGGAGTAT-3′	5′-GCTCTGGTAGGGGCAGTGA-3′
iNOS	5′-GTTCTCAGGCCAACAATACAAGA-3′	5′-GTGGACGGGTCGATGTCAC-3′
IL-12p40	5′-TGGTTTGCCATCGTTTTGCTG-3′	5′-ACAGGTGAGGTTCACTGTTTCT-3′
TGF-*β*1	5′-TGGAGCAACATGTGGAACTC-3′	5′-CAGCAGCCGGTTACCAAG-3′
RANTES	5′-TTTGCCTACCTCTCCCTCG-3′	5′-CGACTGCAAGATTGGAGCACT-3′
CXCL2	5′-CGCCCAGACAGAAGTCATAGCC-3′	5′-TCTTCCGTTGAGGGACAGCAGC-3′
GAPDH	5′-AGGTCGGTGTGAACGGATTTG-3′	5′-TGTAGACCATGTAGTTGAGGTCA-3′
